# Deep rTMS of the insula and prefrontal cortex in smokers with schizophrenia: Proof-of-concept study

**DOI:** 10.1038/s41537-022-00224-0

**Published:** 2022-02-25

**Authors:** Scott J. Moeller, Roberto Gil, Jodi J. Weinstein, Topaz Baumvoll, Kenneth Wengler, Natalka Fallon, Jared X. Van Snellenberg, Sameera Abeykoon, Greg Perlman, John Williams, Lucian Manu, Mark Slifstein, Clifford M. Cassidy, Diana M. Martinez, Anissa Abi-Dargham

**Affiliations:** 1 Renaissance School of Medicine at Stony Brook University, Stony Brook, NY 11794 USA; 2 Columbia University, New York State Psychiatric Institute, New York, NY 10032 USA; 3 University of Ottawa Institute of Mental Health Research, affiliated with The Royal, Ottawa, ON K1Z 8N3 Canada

**Keywords:** Schizophrenia, Neural circuits, Schizophrenia

## Abstract

Patients with schizophrenia have a high prevalence of cigarette smoking and respond poorly to conventional treatments, highlighting the need for new therapies. We conducted a mechanistic, proof-of-concept study using bilateral deep repetitive transcranial magnetic stimulation (dTMS) of insular and prefrontal cortices at high frequency, using the specialized H4 coil. Feasibility of dTMS was tested for disruption of tobacco self-administration, insula target engagement, and insula circuit modulation, all of which were a priori outcomes of interest. Twenty patients completed the study, consisting of weekday dTMS sessions (randomization to active dTMS or sham; double-blind; 10 patients per group), a laboratory tobacco self-administration paradigm (pre/post assessments), and multimodal imaging (three MRI total sessions). Results showed that participants assigned to active dTMS were slower to initiate smoking their first cigarette compared with sham, consistent with smoking disruption. The imaging analyses did not reveal significant Time × Group interactions, but effects were in the anticipated directions. In arterial spin labeling analyses testing for target engagement, an overall decrease in insula blood flow, measured during a post-treatment MRI versus baseline, was numerically more pronounced in the active dTMS group than sham. In fMRI analyses, resting-state connectivity between the insula and default mode network showed a numerically greater change from baseline in the active dTMS group than sham, consistent with a functional change to insula circuits. Exploratory analyses further suggested a therapeutic effect of dTMS on symptoms of psychosis. These initial observations pave the way for future confirmatory studies of dTMS in smoking patients with schizophrenia.

## Introduction

Patients with schizophrenia suffer from substantial disability and premature mortality, worsened by their high rates of tobacco smoking^1^. About 70–85% of individuals with schizophrenia also use tobacco^2^, a prevalence more than five times the general population^3^, driven largely by resistance to quitting^4^. Treatment strategies for tobacco dependence in schizophrenia generally rely on the three standard pharmacotherapies approved by the FDA: nicotine replacement therapies, the norepinephrine/dopamine reuptake inhibitor bupropion, and the nicotinic acetylcholine receptor partial agonist varenicline^5^. Although these treatments provide modest, short-term improvement in smoking outcomes, their efficacy tends to wane over time, and behavioral approaches often fare even worse^6^. As these conventional treatments provide limited optimism for schizophrenia, there is a critical need for new strategies and therapeutic targets.

One potentially viable target for smoking cessation in schizophrenia is the insula. The insula is thought to play a major role in tobacco use disorder with regard to mediating nicotine salience and reward, cigarette craving, and the pleasurable sensations associated with smoking, which motivate and maintain the addictive behavior^7–10^. The necessity of the insula for craving has been demonstrated in stroke patients, where damage to this region produces marked reductions in cigarette urges, withdrawal, and nicotine-seeking behavior^11,12^. The insula is also consistently one of many brain regions that shows robust fMRI activation to smoking cues^13,14^, and its activation is positively correlated with subjective cigarette cravings^15^, nicotine dependence^16^, and smoking relapse after cessation^17^. The mechanism of these relationships may be linked to the insula’s more general role in mediating cognitive and emotional processes such as interoception and decision-making^18,19^. The insula is also a core part of the brain’s salience network, and abnormalities within and between this larger salience network similarly have been associated with smoking behavior. For example, smoking withdrawal has been linked to enhanced insular connectivity with the default mode network (DMN) and salience network^20,21^, and greater connectivity between the insula and larger salience network has been correlated with cigarette craving^21,22^ and fMRI activation to smoking cues^23^. Functional and structural abnormalities of the insula have also been reported in schizophrenia^24,25^, and smoking comorbidity can exacerbate these deficits^26,27^. Interestingly, varenicline has been shown to modulate insula—DMN connectivity^28^, suggesting that this functional pathway could be a viable therapeutic target. These findings support the view that the insula and its functional circuitry may serve as a major therapeutic target for smoking cessation.

One method of modulating insula functioning is through targeted neurostimulation^29–31^, which may be accomplished via deep repetitive transcranial magnetic stimulation (dTMS). dTMS uses a specialized (H4) coil that enables access to deeper brain structures than previously feasible with standard TMS coils, stimulating the bilateral insula along with bilateral ventrolateral and dorsolateral prefrontal cortices (PFC)^32^. In an earlier clinical trial of healthy smokers, high frequency (10 Hz) insula-inclusive stimulation daily for 13 days showed statistically significant effects on smoking-related behavioral measures compared with sham^33^. This therapeutic effect was recently confirmed in a large multisite clinical trial, where 15 sessions of dTMS decreased smoking rates in otherwise healthy smokers for up to several months^34^. This research led to dTMS being granted FDA 510(k) clearance for smoking cessation. The dTMS approach for smoking cessation, however, has not been tested in schizophrenia; previous studies have generally relied on neurostimulation of surface-level cortical areas^35^.

Here, we conducted a mechanistic feasibility study to test whether a 3-week treatment regimen of high frequency, insula-inclusive dTMS bilaterally (versus sham) may disrupt tobacco smoking behavior and modulate insula-centric brain networks in patients with schizophrenia and comorbid tobacco use disorder. Our goal was to provide proof-of-concept for developing future clinical trials with dTMS in this population; as this study was not a full trial and instead was a feasibility study, we were not asked by the funding Institute to preregister. Nonetheless, all our outcome measures were pre-specified in the R21 mechanism that supported the study. We hypothesized that: (1) daily dTMS over 3 weeks would reduce the choice of tobacco in a self-administration paradigm compared with sham (latency to start smoking and number of cigarettes smoked); (2) daily dTMS over 3 weeks would induce a change in arterial spin labeling (ASL) measured blood flow of the insula, in at least one of the two post-treatment MRI scans (acute and at 3 weeks) compared with sham; and (3) daily dTMS over 3 weeks would induce a pre-post treatment change in insula-centric networks compared with sham. For the latter, our a priori interest was bilateral insula connectivity with the DMN, which was previously shown as a functional target for smoking therapeutics including nicotine and varenicline administration^28^. More recently, a study of healthy individuals showed that a single session of insula-inclusive dTMS, delivered at either high or low frequencies, modulated resting-state connectivity between the insula and medial PFC (part of the DMN)^36^. On an exploratory basis in our study, we also examined the effects of dTMS on symptoms of schizophrenia, using the Positive and Negative Syndrome Scale (PANSS)^37^.

## Results

### Methods Overview

**Fig. 1:**
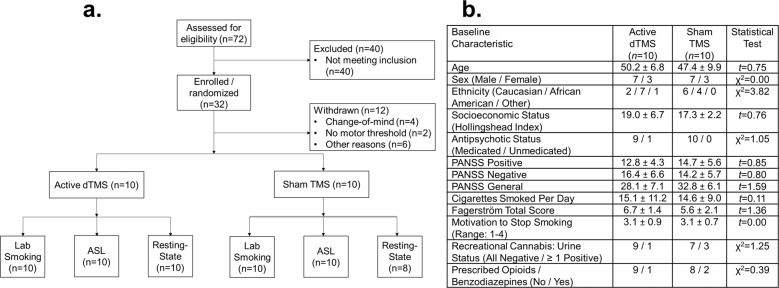
Study participants. **a** Flow diagram for the trial. ASL = arterial spin labeling. **b** Demographics of patients who completed the study. For **b**, numbers are frequencies or *M* ± SD. dTMS = deep repetitive transcranial magnetic stimulation, PANSS = Positive and Negative Syndrome Scale. For additional description of the Fagerström scale, motivation to stop smoking, and cigarettes smoked per day (the latter assessed with a Timeline Follow-Back calendar), see the Supplement. Socioeconomic status was measured with the Hollingshead Index^66^. No statistical tests showed significant differences between the treatment groups.

**Fig. 2:**
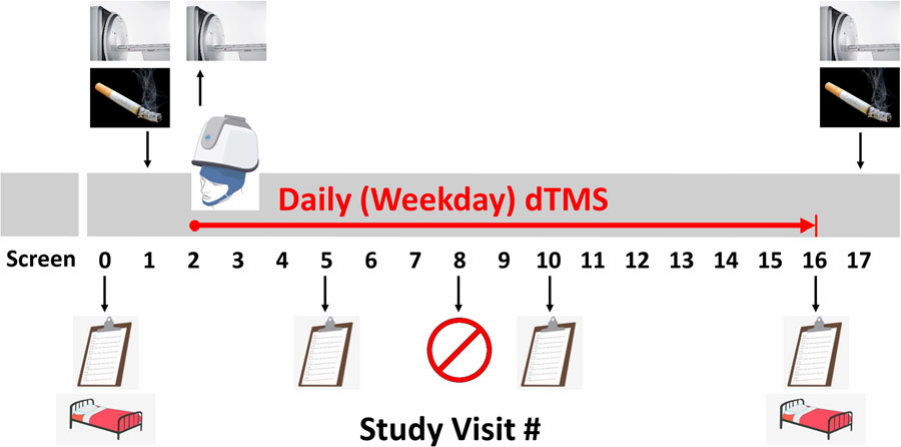
Overview of study procedures. The study included pre- and post-treatment smoking self-administration sessions (cigarette graphic), which were preceded by overnight inpatient stay to monitor smoking abstinence (bed graphic); weekday deep repetitive transcranial magnetic stimulation (dTMS) treatment for 15 weekdays over 3 weeks (Visits 2-16; TMS helmet graphic), and three functional MRI scans (MRI scanner graphic), during which ASL was acquired (once at baseline, once after the first treatment, and once after the final treatment). MRI was acquired before the smoking self-administration on Days 1 and 17, and after the first dTMS session on Day 2. Resting-state functional connectivity was also acquired during the same MRI sessions, but only during the first and last scan (Visits 1 and 17). Clinical assessment with the PANSS was acquired at four time points (clipboard graphic), approximately once per week. Participants made a quit attempt approximately by Visit 8 (red prohibition graphic). Screening/consenting could occur up to a week before primary study procedures began.

We screened 72 patients from June 2017 to January 2020, enrolling 32 of them (Fig. 1). Diagnosis was confirmed by SCID-5^38^, completed during the baseline assessment (Visit 0). Of these 32 enrolled patients, 20 completed a 3-week dTMS treatment and all relevant study procedures (*N* = 10 active dTMS; *N* = 10 sham dTMS; 17 total Visits) (Fig. 2). No participant missed more than one dTMS session. Twelve patients were withdrawn for the following reasons: (A) withdrawal of consent (*n* = 4); (B) inability to detect a motor threshold (MT) for the delivery of the dTMS (*n* = 2); (C) positive urine drug screen for illicitly-used substances (*n* = 2); (D) MRI contraindications (*n* = 3); and (E) legal problems that would have precluded participation in a multi-week, multi-visit study (*n* = 1).

We analyzed the following outcomes of interest, all defined a priori (see “Methods” for complete information): tobacco self-administration (latency to smoke and number of cigarettes), blood flow in the bilateral insula measured with ASL, and resting-state functional connectivity between the bilateral insula and DMN (extracted correlation values of the fMRI time series). Analyses were performed with generalized estimating equations (GEEs), examining for changes over time (Visit) as a function of treatment assignment (Group). Table 1 displays the statistics for these study outcomes. Symptoms of schizophrenia were acquired with the PANSS and analyzed on an exploratory basis (fully described in the Supplement).

**Table 1:** Deep rTMS (dTMS) effects on primary variables: Smoking behavior, insula blood flow, and insula-centric connectivity.

Regressor	*M*_diff_ (SE)	*p* value	Source of effect or trend
Smoking behavior: Probability first cigarette within 5 min			
Visit (ref = Visit 1)			
Visit 17	−0.26 (0.27)	0.327	NA
Group (ref = Sham)			
Active dTMS	−0.26 (0.56)	0.642	NA
Visit × Group			
Visit 17 × Active dTMS	−1.16 (0.54)	0.030*	Active dTMS: Visit 17 < Visit 1; Sham: Visit 17 = Visit 1
Smoking behavior: Number of cigarettes smoked			
Visit (ref = Visit 1)			
Visit 17	0.00 (0.22)	1.000	NA
Group (ref = Sham)			
Active dTMS	−0.00 (0.57)	1.000	NA
Visit × Group			
Visit 17 × Active dTMS	−0.60 (0.44)	0.170	NA
Cerebral blood flow: Bilateral insula			
Visit (ref = Visit 1)			
Visit 2	−3.67 (1.72)	0.033*^a^	Visit 2 < Visit 1
Visit 17	−1.89 (1.46)	0.198	NA
Group (ref = Sham)			
Active dTMS	−4.50 (3.03)	0.137	NA
Visit × Group^b^			
Visit 2 × Active dTMS	−0.90 (3.44)	0.794	NA
Visit 17 × Active dTMS	3.06 (2.93)	0.296	NA
Resting-state functional connectivity: Bilateral insula seed with default mode network			
Visit (ref = Visit 1)			
Visit 17	0.03 (0.04)	0.497	NA
Group (ref = Sham)			
Active dTMS	−0.05 (0.07)	0.503	NA
Visit × Group			
Visit 17 × Active dTMS	0.13 (0.09)	0.122†^a^	Active dTMS: Visit 1 numerically lower than Visit 17; Sham: Visit 1 = Visit 17

Note. *p < 0.05, †*p* < 0.15.

^a^Further tested with posthoc comparisons on an exploratory basis, as explained in the main text.

^b^Omnibus Visit × Group interaction: χ^2^(2) = 2.22, *p* = 0.329.

### Smoking self-administration

**Fig. 3:**
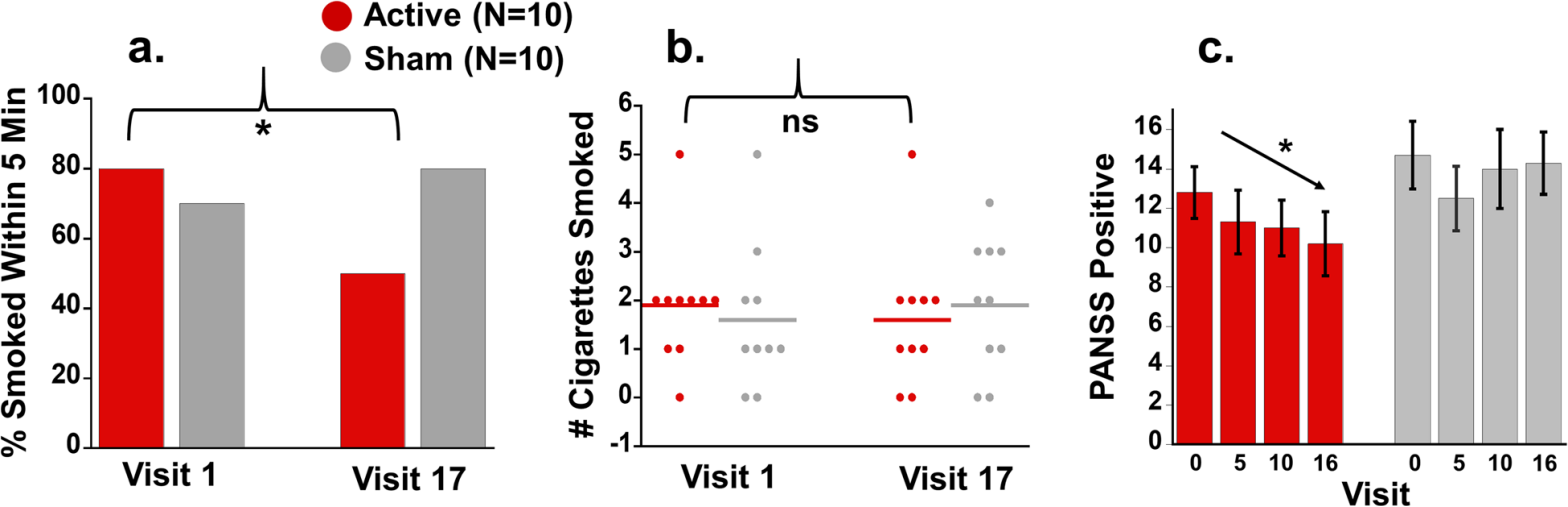
Effects of dTMS on behavior. **a**, **b** In a laboratory task of tobacco choice, participants had the chance to smoke or earn money for abstaining. **a** There was a significant interaction on the latency to smoke a first cigarette, where patients in the active dTMS group took longer to smoke after treatment than before it (significant effect of Visit in this group indicated by asterisk). **b** While there was a similar direction of effects regarding the total number of cigarettes smoked during the task, no main effects or interactions were significant (ns = not significant). In addition to these smoking variables, **c** dTMS improved severity of psychosis, as measured by the Positive and Negative Syndrome Scale (PANSS). Uniquely in the active dTMS group, there was a linear decrease in positive symptoms over the course of the study (significant linear contrast indicated by asterisk).

For the latency to first cigarette, results showed a significant Visit × Group interaction as hypothesized (*p* = 0.030, partial *η*^2^ = 0.206) (Table 1). The interaction was driven by a lower probability of immediate smoking on Visit 17 (post-treatment) than Visit 1 (pretreatment), after active dTMS treatment (*b* = −0.30, SE = 0.15, *p* = 0.044) but not after sham dTMS (*p* = 0.30) (Fig. 3a). That is, dTMS patients after treatment were slower to initiate smoking when given the opportunity. In contrast, number of cigarettes smoked showed no treatment effects (Fig. 3b) (Table 1).

### Exploratory analysis: Symptoms of schizophrenia

All participants had more PANSS Positive (psychotic) symptoms at baseline (Visit 0) than at subsequent (approximately weekly) Visits (main effect) (see Supplementary Table 2, for statistics). This main effect was qualified by a significant Visit × Group interaction, which revealed differences between Visit 0 (pretreatment) and Visit 16 (post-treatment) as a function of Group (*b* = −2.20, SE = 0.99, *p* = 0.026, partial *η*^2^ = 0.216). Follow-up linear contrasts showed a stepwise reduction of positive symptoms across the four time points in the active dTMS group (*b* = −0.91, SE = 0.36, *p* = 0.011), but not sham (*p* = 0.84) (Fig. 3c). Negative and General PANSS subscales did not show the same pattern (Supplement; Supplementary Table 2).

### Insula blood flow

**Fig. 4:**
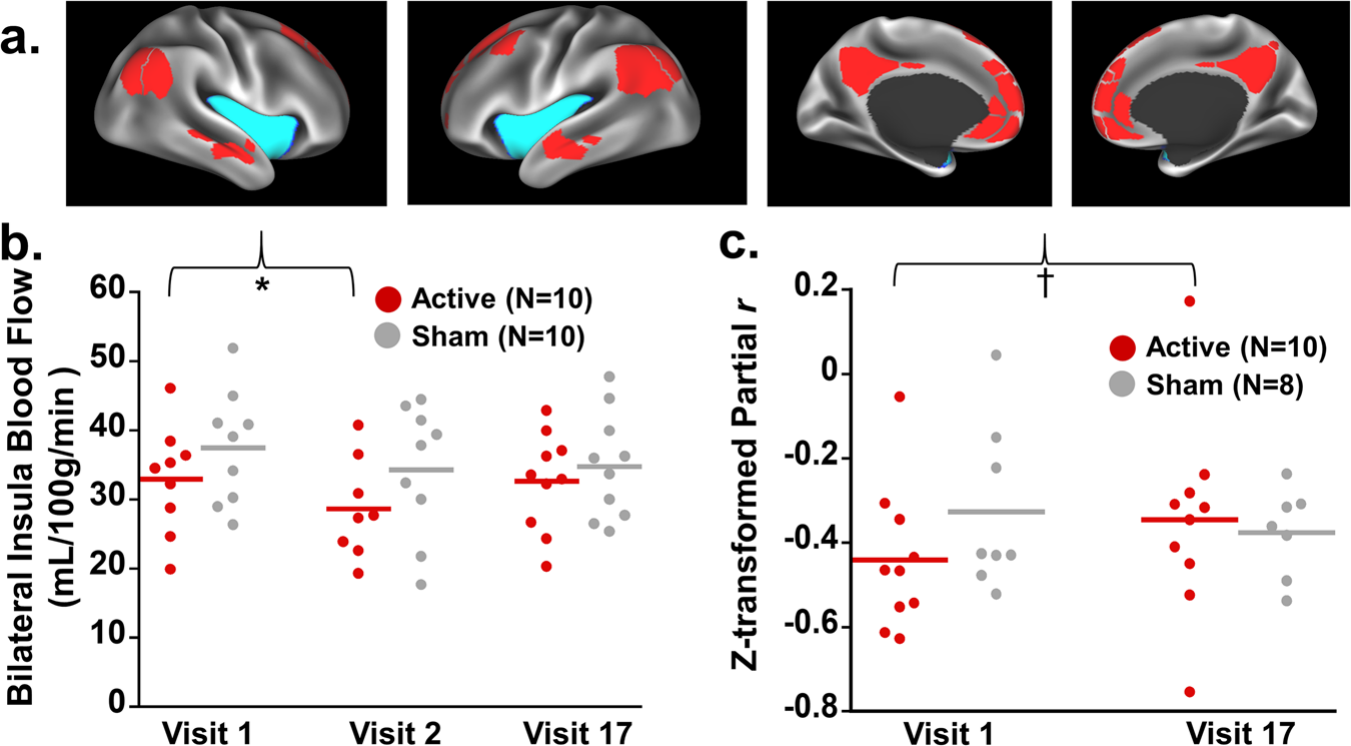
Effects of dTMS on insula-centric neural functioning. **a** Regions of interest (ROIs) for the insula (teal) and the default mode network (DMN) (red). **b** Both groups showed a decrease in insula cerebral blood flow (CBF) during Visit 2, which occurred directly after the first dTMS treatment. Exploratory analyses suggested that this decrease during Visit 2 was significant in the active dTMS group (indicated by asterisk), but not the sham group, consistent with a more robust numerical difference between baseline and first treatment in those receiving the active stimulation. **c** The active dTMS group showed numerically increased (less negative) insula-DMN connectivity from Visit 1 to Visit 17 (*p* < 0.15, indicated by †), which was not evident in the sham group. The *y*-axis depicts the *z*-transformed partial *r*, between the bilateral insula and the DMN (with the insula as the seed).

Figure 4a displays the insula ROI. There were no significant Visit × Group interactions. However, across all participants, insula CBF was lower at Visit 2 (first post-treatment MRI) than Visit 1 (pre-treatment MRI) (Fig. 4b; Table 1). Insula CBF at Visit 17 did not differ from Visit 1. In exploratory analyses, Visit 2 differed from Visit 1 in the active dTMS group (*b* = −4.11, SE = 2.09, *p* = 0.0495) but not the sham group (*p* = 0.24), further evidenced by a quadratic contrast which was significant in the active dTMS group (*b* = 1.86, SE = 0.80, *p* = 0.021) but not the sham group (*p* = 0.51). Thus, exploratory analyses suggested that the overall reduction in insula CBF after the first dTMS session was mostly attributable to the active dTMS group as expected. By the last Visit, however, the groups were comparable.

### Insula-DMN functional connectivity

Figure 4a displays ROIs for the insula and DMN. Results with resting-state connectivity did not reach conventional significance levels [Visit × Group interaction: *p* = 0.12, partial *η*^2^ = 0.130) (Fig. 4c; Table 1)]. Still, in exploring the data, the active dTMS group had numerically increased (less negative) insula-DMN connectivity (*b* = 0.10, SE = 0.06, *p* = 0.11), whereas the sham group did not (*p* = 0.54). These observations may inform future research.

### Adverse events

There were 12 discrete adverse events during the trial, reported by six patients (5 active, 1 sham). In the active dTMS group, adverse events included head/facial discomfort (*n* = 3), tingling/twitching in the hands (*n* = 3), back pain (*n* = 2), and feeling that the stimulation was too powerful (*n* = 2); there was also one report of an accidental fall outside the lab, which was not serious. In the sham group, there was a single report of neck/chest discomfort during the last minute of one session. Importantly, all adverse events were minor and were resolved without sequelae, and none resulted in study withdrawal.

## Discussion

We conducted a proof-of-concept study testing the capability of dTMS of the insula and PFC to disrupt tobacco self-administration in patients with schizophrenia. Consistent with our hypotheses, 3-week treatment with dTMS significantly increased the latency for patients to smoke their first cigarette during an ecologically-valid, experimentally-controlled tobacco choice paradigm^39^, suggesting reduced motivation to smoke during withdrawal. Exploratory analyses with self-reported craving and smoking satisfaction largely corroborated this interpretation (Supplement). We did not detect a significant effect on the total number of cigarettes smoked during the task, though the direction was similar to the latency effects. One potential explanation is that, once participants smoked their initial cigarette to quell acute withdrawal and craving, subsequent smoking during the task may have been driven by ancillary factors such as alleviating boredom^40^, thereby reducing the magnitude of a treatment effect. Although our results in totality must be considered preliminary given the small sample size, the significant effect on latency to smoke lends support for initiating clinical trials aimed at testing dTMS for smoking cessation in schizophrenia.

In parallel to the behavioral effects, ASL analyses showed decreased insula CBF after the first session, a significant main effect that was largely driven by the active dTMS group. This finding provides initial indication of target engagement. Insula perfusion was previously reported to correlate positively with abstinence-induced cigarette craving^41^, suggesting that CBF reductions with treatment could be desirable. By the end of treatment, however, insula CBF had returned to baseline levels, perhaps reflecting acclimatization to the stimulation over time^42^. Even so, the initial evidence of an ASL signal during at least one dTMS session supports the potential viability of this neurostimulation approach for future therapeutic applications, indicating an ability to reach the insula with sufficient power^43^. Our use of ASL as an index of dTMS target engagement also supports and extends prior research in schizophrenia. In a previous study, rTMS treatment delivered to the temporoparietal cortex decreased blood flow in this same region, which correlated with a decrease in auditory verbal hallucinations^44^. In our study, correspondingly, a linear decrease over time in PANSS positive symptoms could be partially due to stimulation that, while aimed at targeting the insula, may have also affected portions of the temporoparietal cortex. However, for reasons of sample size, we elected here to restrict analyses to the insula which was hypothesized a priori. We also acknowledge an interpretational constraint with these ASL analyses in that, across the whole sample, cigarette abstinence lengths differed between the first and second MRIs, whereas abstinence lengths for the first and third MRIs did not differ (see “Methods”). Importantly, though, this constraint could not explain why the ASL decrease appeared to be more pronounced in the active group (i.e., given that the groups did not differ at baseline). Thus, the ASL results are consistent with a treatment signal reflecting target engagement, but need to be confirmed in future studies.

In our final a priori measure of interest, we observed a numerical change in insula-centric network connectivity with dTMS. More specifically, insula-DMN connectivity in the active group, which was initially highly negative (anticorrelated), became less anticorrelated after active dTMS treatment, albeit not significantly so. Previous studies in healthy individuals have placed the insula within the cingulo-opercular task-control network, which is negatively correlated with the task-negative (including DMN) network under normal circumstances^45^, therefore, supporting the overall (negative) direction of the connectivity values observed in our study. Insofar as more efficiently tuned interactions between these respective task-control and task-negative networks have correlated with better task performance^46^, we initially speculated that active dTMS treatment might render insula-DMN connectivity more anticorrelated. One potential explanation for numerical effects in the opposite direction is that a more negative insula-DMN functional circuit as observed in the sham group may reflect a state of enhanced craving for tobacco during smoking abstinence^47^, which was then partially alleviated by dTMS. Furthermore, connectivity within and between the salience network, which includes the insula, is often weakened in schizophrenia relative to healthy controls^48^. In particular, when the insula is examined as a seed region in resting-state studies, studies have reported reductions between the insula and diffuse cortical and subcortical regions across the brain including regions comprising the DMN^49^. In some cases, the extent of disrupted connectivity in schizophrenia has been correlated with hallucination severity^50^, social withdrawal^51^, and cognitive deficits^24^. Insula dysconnection can be rescued with antipsychotics^52^. Thus, given that insula connectivity with other brain networks is weakened in schizophrenia, it is possible that a strengthening of this circuit could be therapeutically desirable. Nevertheless, as with the observations from this study, larger sample sizes are needed.

This study has several limitations. The primary limitation is the small sample size, meaning that our results should be considered preliminary, particularly the neuroimaging for which Visit × Group interactions were not significant. Our study was the first step required to take on larger future investigations. Second, while we used a largely similar dTMS protocol as a recent clinical trial in healthy smokers^34^, we note that one major difference is that our study did not include a craving provocation, because the importance of doing so was not known when our study was initiated. Importantly, though, the lack of a pre-session provocation stands to work against our hypotheses of finding a treatment signal, meaning that the effect sizes reported here, in fact, may be underestimating the true therapeutic potential of this modality, providing some optimism for future studies. Such future studies indeed should incorporate a pre-session provocation, which may increase the measurable therapeutic effect. Third, as mentioned above, the first post-treatment MRI differed from the first MRI not only for effects of dTMS but also smoking abstinence versus satiety. Future studies will need to standardize scanning according to the time since last cigarette. Fourth, while the insula consists of several functionally distinct anterior-to-posterior subregions^53^, we analyzed the whole region together, due to concerns about power and multiple comparisons. Fifth, our patient sample was relatively homogeneous, comprising mostly middle-aged patients treated with antipsychotics (Fig. 1b). It remains to be determined whether these effects generalize to younger patients. However, this limitation may also be viewed as a strength, in that dTMS served to complement standard-of-care pharmacotherapies^54^. Sixth, the durability of these effects, including long-term effects on daily smoking, are unknown. Answering such questions will require well-resourced clinical trials with longitudinal follow-ups. Seventh, because dTMS also stimulates cortical regions dorsal to the insula, stimulation of these other regions may have contributed to some of the therapeutic signal seen here. While the neural data show feasibility that the insula was reached, we did not have the power or the experimental design to dissect the relative contributions of the insula versus surface PFC regions. Finally, we did not systematically probe participants’ beliefs about experimental group assignment. This was done to maximize experimenter blinding, but clinical trials building on these results could consider adopting this practice.

In conclusion, despite a small sample, we provide proof-of-concept that insula-inclusive dTMS may disrupt smoking behavior and modulate insula-centric neural function in patients with schizophrenia. The next step will be to conduct a clinical trial testing the efficacy of dTMS on prospectively-measured cigarette smoking, craving, and abstinence, while also replicating and clarifying our observed effects on the underlying neurobiology and clinical symptoms. Such a project can advance an exciting new approach to smoking cessation in schizophrenia, to help patients conquer their nicotine addiction and improve their overall longevity and quality-of-life.

## Methods

### Participants

This study was approved by the Stony Brook University Institutional Review Board (IRB). All study participants provided written informed consent. Capacity to consent was confirmed by a clinician unaffiliated with the study, using the MacArthur Competence Assessment Tool for Clinical Research (MacCAT-CR)^55^. Inclusion criteria were: (A) 18–60 years old; (B) DSM-5 criteria for schizophrenia or schizoaffective disorder; (C) DSM-5 diagnosis of tobacco use disorder and expressed desire to quit (without regard for number of cigarettes smoked per day, to enhance generalizability and feasibility of recruitment); (D) negative urine toxicology for illicitly-used substances, other than cannabis; and (E) english fluency, for completion of study materials. Exclusion criteria were: (A) clinically-significant psychopathology other than schizophrenia or schizoaffective disorder; (B) substance use disorder within the past 12 months, except for tobacco; (C) current use of smoking cessation medications or products; (D) change in psychotropic medication in the last 4 weeks; (E) hospitalization in the last 3 months; (F) history of suicidal or homicidal tendencies; (G) history of epilepsy; (H) Clinical Global Impressions (CGI) rating of 6 (severely ill) or 7 (extremely ill); (I) pregnancy or lactation (females only); (J) lack of effective birth control (females only); (K) contraindications to MRI; (L) lack of capacity to give informed consent; and (M) prisoner status. During weekly toxicology tests, seven participants produced ≥ 1 (*M* = 2.23 ± 1.38) positive urine tests for a potentially addictive substance. Of those seven, three had valid prescriptions for the substance meaning that its use was not illicit (i.e., for clonazepam, suboxone, and oxycodone), while the other four participants used cannabis recreationally; neither of these differed between the treatment groups (Fig. 1b). Non-prescribed cannabis use in the previous month was originally exclusionary, but was changed due to the relaxing of criminal penalties in New York State. No participants used alcohol excessively during the study. Most participants were currently taking antipsychotics (Fig. 1b), which were maintained at the same doses throughout the study. Participants were instructed to make a best-effort cigarette quit attempt by Visit 8 of the study (see Supplement for results showing that all participants reduced their smoking during the trial). Figure 2 provides a schematic of the procedures completed by the study participants.

### dTMS procedures

Treatment with dTMS occurred each weekday for three weeks, with the exception of holidays, using the system provided by Brainsway (Brainsway, LTD, Jerusalem, Israel). The dTMS protocol used in this study was largely consistent with the one described in Zangen and colleagues^34^, a comprehensive clinical trial of dTMS in healthy smokers. One critical difference, however, is that our study did not include a pre-dTMS craving provocation (see Limitations). Each dTMS session (for active and sham groups) was conducted as follows. First, we determined the position of the right abductor pollicis brevis (APB) motor cortex, finding the minimal MT required for its activation, which determined the strength of the dTMS pulses. To measure MT, the APB was stimulated in the hand area of the motor cortex (one pulse every 5 s), with the MT defined as the lowest intensity of stimulation producing visually-perceptible movement of the finger in >3 of 6 trials. The MT was determined, and adjusted if needed, once per week for each participant.

After determining the MT, dTMS stimulation was applied 6 cm anterior to the motor “hot spot” bilaterally (using ruler cap for positioning), at 120% of the MT. The target threshold was reached gradually over the first three sessions, allowing time for patients to acclimate. At the beginning of each treatment session, the operator applied one short trial train of 0.5 s, 10 Hz, at 120% of the MT, to ensure that the coil was in position and the treatment was tolerable. During the first treatment, participants received stimulation at 100% of the MT. During the second treatment, stimulation intensity was increased to 110% of the MT. Beginning at the third treatment and continuing onward, participants received a treatment at 120% of the MT. Each treatment consisted of 60 trains, each lasting 3 s and interleaved with a 15 s delay, delivered over 20 min.

The sham coil, which was used as the experimental control, mimicked the real coil’s auditory artifact and scalp sensations. It also produced similar activation of facial muscles, without stimulating the brain itself. Magnetic cards were inserted into the machine to control its operating mode (i.e., whether a participant was to receive active or sham treatment). Participants were randomized upon enrollment, stratified by gender, number of cigarettes smoked per day (>10, <10), and age (<32, 32–45, 46–60)^56^. Active and sham magnetic cards did not differ in appearance, and both coils were enclosed within the same treatment helmet, together enabling double-blind administration. Only one coauthor, who oversaw the randomization and had no further contact with the participants, knew the treatment assignment for each participant while the study was ongoing. As this was a small pilot study that lacked the resources for an independent data analyst who was sufficiently detached from other study procedures, we could not feasibly employ triple-blinding.

### Tobacco choice (self-administration) sessions

To obtain a precise and controlled measure of the therapeutic potential for dTMS in this feasibility trial, we used the laboratory-based McKee Smoking Lapse Test^39^. This unique paradigm involves overnight nicotine deprivation leading to withdrawal (which was accomplished by overnight stay in the hospital; see Fig. 2), time to first cigarette upon its availability (up to 50 min in duration, ending upon smoking initiation), and a subsequent *ad libitum* self-administration period (60 min in length, which began as soon as smoking was initiated). The paradigm yielded two dependent measures. The first measure was latency (minutes) to first cigarette after overnight abstinence (i.e., ability to resist smoking in the midst of nicotine withdrawal). Given that latency was found to follow a bimodal distribution, we dichotomized this variable into whether (yes/no) participants smoked within the first 5 min of beginning the task, so that it could be feasibly analyzed. The second measure was the total number of whole cigarettes smoked over the next 60 min during the *ab libitum* period. Participants could earn up to $14 if they fully abstained for a total of 110 min; compensation was adjusted downward depending on how quickly and how many cigarettes were smoked. Participants completed this task twice, before the first dTMS treatment (Visit 1) and after the final treatment (Visit 17).

### Neuroimaging procedures

We acquired ASL to measure insula CBF as an index of target engagement, and resting-state connectivity to measure modulation of insula-centric networks. MRI data were acquired before the first treatment (Visit 1: both ASL and connectivity acquired), after the first dTMS treatment (Visit 2: only ASL acquired), and after the final dTMS treatment (Visit 17: both ASL and connectivity acquired). MRI was acquired before the smoking self-administration on Days 1 and 17, and after the first dTMS session on Day 2. In this way, we note that the Day 2 MRI was completed under conditions of relative smoking satiety, in comparison to Days 1 and 17 which were under conditions of smoking deprivation (i.e., the days after participants stayed overnight in the hospital). Importantly, though, smoking abstinence versus satiety, which was the same for all participants, does not confound any observed dTMS treatment effects between the groups, which were created through random assignment.

Scanning was performed on a 3T Prisma^Fit^ MRI (Siemens, Erlangen, Germany) using a 64-channel head coil. T1-weighted (T1w) 3D MPRAGE images were acquired with the following parameters: repetition time (TR)/echo time (TE)/inversion time (TI) = 2400/2.24/1060 ms, flip angle (FA) = 8°, matrix size = 320×320×208, 0.8 mm isotropic resolution, and GRAPPA parallel imaging factor = 2. T2-weighted (T2w) 3D SPACE images were acquired with the following parameters: spatial resolution = 0.8 mm isotropic resolution, matrix size = 320×320×208, TR/TE = 3200/564 ms, echo spacing = 3.86 ms, echo train duration = 1166 ms, variable flip angle (T2 var mode), and GRAPPA parallel imaging factor = 2.

For ASL, 3D-GRASE pseudo-continuous arterial spin labeling (pCASL) images were acquired with the following parameters: TR/TE/label time/post label delay (PLD) = 4000/17.6/1600/1700ms, FA = 120°, matrix size = 80×80×40, 3 mm isotropic resolution, GRAPPA parallel imaging factor = 2, phase-encoding direction segmentation factor = 2, partition direction segmentation factor = 3, and number of control-label averages = 12. Two background suppression pulses were applied to suppress ~90% of static tissue signal. Equilibrium magnetization images were acquired with the same imaging parameters except TR = 8000 ms and no background suppression.

ASL images were processed using SPM12 and in-house customized ASLtoolbox^57^. The processing pipeline consisted of: realignment for motion correction; segmentation of the T1w image into gray matter (GM), white matter (WM), and cerebrospinal fluid (CSF) probability maps; coregistration of the T1w image, GM, WM, and CSF probability maps to ASL images; smoothing with a 3 mm full-width at half maximum (FWHM) isotropic Gaussian kernel; CBF quantification using the single-compartment model^58^ with 3D partial volume correction^59^; and coregistration of the partial volume corrected CBF map to the T1w image for region-of-interest (ROI) analysis. ROIs were generated from the T1w images using FreeSurfer^60^. All 20 participants contributed usable data. Out of 60 possible ASL data points (20 participants × 3 Visits), 55 were usable.

For resting-state connectivity, four runs of multiband^61^ blood-oxygenation-level-dependent (BOLD) sensitive echo-planar imaging (EPI) T2*-weighted resting-state imaging were acquired per session. Each run comprised 563 volumes, approximately 7.5 min per run. During acquisition, participants fixated on a white cross. Phase encoding direction (i.e., anterior–posterior and posterior–anterior) was alternated for each run. The following acquisition parameters were used: multiband acceleration of 6 and no GRAPPA, 2 mm isotropic, 204 mm FOV, 66 slices, 60° FA, and TR/TE = 800/25 ms (for complete information, see the Supplement). Data were preprocessed using the following Human Connectome Project (HCP)^62^ Minimal Preprocessing Pipelines v4.2 (https://github.com/Washington-University/HCPpipelines) (for complete information, see the Supplement), smoothed with a 2 mm FWHM Gaussian kernel.

For resting-state analysis, the DMN was identified using the cortical parcellation and partition developed by Gordon and colleagues using resting-state correlations^63^, and the average DMN signal was extracted for each participant as the mean BOLD signal across all DMN parcels. Left and right insula were identified from the FreeSurfer cortical parcellation for each subject using the Desikan-Killiany cortical atlas^64,65^, and average insula signal was obtained as the mean signal across both insula parcels (same insula ROIs as for the ASL). Insula-DMN functional connectivity was then calculated in each run as the partial correlation between mean time series after volume censoring (see Supplement for details), and after controlling for the following 30 nuisance parameters: 6 band-pass filtered motion parameters (fMPs) and their squares, the derivatives of the 6 fMPs and their squares, CSF signal and its derivative, WM signal and its derivative, and global signal and its derivative. Partial correlations were Fisher’s z-transformed and averaged across runs within each session. Out of 40 connectivity data points, 35 were usable from 18 participants; the two participants who had to be excluded for poor data quality were both from the sham group.

### Symptoms of schizophrenia

On an exploratory basis, we examined the PANSS^37^, acquired four times by trained raters with established interrater reliability. Assessments took place at baseline (Visit 0), and then approximately weekly (Visits 5, 10, and 16).

### Statistical analyses

We performed a series of GEEs, which are more robust to missing data than mixed ANOVA and can accommodate categorical outcomes. In these models, Visit was a within-person factor (typically 2 levels comprising before and after treatment, but up to 4 levels for some variables, such as the PANSS which was administered four times); Group (active, sham) was a between-person factor. Time to first cigarette, treated as a categorical variable due to the dichotomization described above, was modeled with a binomial distribution (probit link function, exchangeable covariance structure, and robust standard errors). All other (continuous) dependent variables were modeled with a Gaussian distribution (identity link function, exchangeable covariance structure, and robust standard errors). We were primarily interested in the Visit × Group interactions, testing for changes in the dependent variables over time as a function of receiving active versus sham dTMS (though main effects were also interpreted if appropriate). Significant interactions were followed with posthoc comparisons to localize their source; variables with more than two Visits (ASL, PANSS) were also examined with linear and quadratic contrasts where applicable, to examine trajectories over time. Significance was set at *p* < 0.05, though effects up to *p* < 0.15 were inspected on an exploratory basis. Effect sizes (partial *η*2) were estimated based on chi-square statistics for Visit × Group interactions reaching at least *p* < 0.15 (two-tailed). While we acknowledge that *p* < 0.15 is a lenient threshold for exploring the data, our main goal in this proof-of-concept study was to provide initial observations that may guide future research—a goal that required a comprehensive examination of all potential effects and numerical trends. This was particularly important for the imaging data, to offer a suggestion regarding the potential mechanism of the dTMS. Data were analyzed using Stata MP (v14.2). One set of interim analyses was conducted prior to those reported here, to generate a progress report per requirements of the funding Institute.

### Reporting summary

Further information on research design is available in the Nature Research Reporting Summary linked to this article.

## Supplementary information

Supplementary Material

REPORTING SUMMARY

## Data Availability

The data that support the findings of this study are available from the corresponding authors upon reasonable request.
