# Supplementary material for: Deep rTMS of the insula and prefrontal cortex in smokers with schizophrenia: Proof-of-concept study
Source: Schizophrenia (Heidelb). 2022 Feb 25;8(1):6. doi: 10.1038/s41537-022-00224-0 (PMC8881463; doi:10.1038/s41537-022-00224-0)

SUPPLEMENTARY METHODS

Baseline Smoking Dependence Measures

At baseline (Visit 0), participants completed the Fagerstrom Test for Nicotine Dependence[1], an extensively validated and widely-used measure of smoking severity; responded to a single-item assessment of their motivation to quit smoking [“How determined are you to give up smoking at this attempt?”, scored from 1 (“*Not all that determined*”) to 4 (“*Extremely determined*”)]; and reported the number of cigarettes smoked per day over the past 30 days using a Timeline Follow-Back Calendar[2]. As expected with randomization, these baseline measures did not differ between the experimental groups (Figure 1B, main text). The mean motivation to quit in the sample was 3.10 ± 0.79, reflecting high motivation.

Exploratory Questions during Tobacco Choice

Participants completed the Questionnaire on Smoking Urges[3]. Given the insula’s role in cigarette craving, we analyzed the scale’s Factor 1 (reflecting a strong desire and intention to smoke). Also, given the insula’s role in interoception, we acquired and analyzed a single item from the Modified Cigarette Evaluation Questionnaire[4], which asked, “Did you enjoy the sensations in your throat and chest?” These questionnaires were assessed concurrently with the tobacco choice sessions (Visits 1 and 17).

Quit Attempt

Participants were asked to stop smoking by Visit 8. The Timeline Follow-Back Calendar was assessed at baseline (Visit 1) and again after the study’s conclusion (Visit 17), to obtain information on whether participants were able to stop or reduce smoking. Specifically, we analyzed number of cigarettes smoked per day during the week before

the first treatment compared with the number of cigarettes smoked during the week before the final treatment. Data were missing from one participant in the active dTMS group.

Supplementary Resting-State fMRI Methods

Throughout the resting-state functional scans, a member of the study team manually monitored the display of an eye-tracking camera (which was not recording), to detect and record sustained eye-closures in each participant. The timing, relative to the start of each functional run, of the beginning and end of each eye closure was recorded at the time of the scan, and used to exclude data acquired while participants' eyes were closed.

In accordance with Human Connectome Project (HCP) processing[5], in addition to the resting-state scans we acquired a B0 field map and two brief (3 volume) spin echo images, one in each phase encode direction (anterior-posterior and posterior-anterior). Geometry of these acquisitions matched the multiband EPIs to allow for correction of magnetic field distortions in the EPI images. Structural and functional images were converted from DICOM to NIfTI-1 format using dcm2nii (https://people.cas.sc.edu/rorden/mricron/dcm2nii.html). Data were preprocessed using HCP Minimal Preprocessing Pipelines v4.2 (https://github.com/Washington-University/HCPpipelines)[6], with full methods described previously [7]: (A) *PreFreeSurfer*, (B) *FreeSurfer*, (C) *PostFreeSurfer*, (D) *fMRIVolume*, and (E) *fMRISurface*. Slice timing correction was disabled and surface registration completed using cortical surface matching (MSMsulc)[8,9]. Susceptibility Distortion Correction (SDC) was completed using the spin echo field maps acquired for each participant. Dependencies included the

FMRIB Software Library (FSL)[10,11] v6.0.3, FreeSurfer[12] v6.0.0, and Connectome Workbench v1.4.2 (https://github.com/Washington-University/workbench). HCP preprocessed Blood-Oxygenation-Level-Dependent (BOLD) time series for each run were obtained in the 91k CIFTI (Connectivity Informatics Technology Initiative) format, comprising two cortical surfaces on the 32k_fs_LR (Conte69) greyordinate mesh (average spacing of approximately 2 mm) and a subcortical volume in Montreal Neurological Institute (MNI) 152 non-linear 6th-generation space (MNI152NLin6)[13] with 2mm isotropic voxels. Additionally, 4D volumetric functional images were acquired for each run, also with a spatial resolution of 2×2×2 mm and in MNI152NLin6 space.

Resting-State Connectivity Post-Processing

Nuisance signals for global signal (GS), white matter (WM) signal, and cerebrospinal fluid (CSF) signal were obtained for each run using 4D volumes and compartments defined by the FreeSurfer parcellation[14,15]. GS was calculated as the mean signal over all brain voxels; WM signal and CSF signal were determined by iteratively eroding each compartment (in 3D) until further erosion would result in zero voxels remaining, for a maximum of four erosions[16].

Extracted time series were mode 1000 normalized (multiplied by 1000 and divided of the modal value of all in-brain voxels), linearly detrended, and mean centered, followed by 0.009 to 0.08 Hz band-pass filtering using a second-order zero-phase Butterworth filter. After filtering, the first and last 30 volumes of each time series were discarded, due to contamination by edge artifacts produced by band-pass filtering.

Framewise Estimates of Participant Motion and Signal Fluctuation

Motion parameters (MPs) were obtained from the *fMRIVolume* pipeline as the estimated net translation and rotation between frames using a rigid body (6 degree of freedom) realignment procedure using FSL's FMRIB's Linear Image Registration Tool (FLIRT)[17,18]. MPs were then low-pass filtered using 0.2 Hz zero-phase second-order Butterworth filter; filtered rotation MPs were subsequently converted to the estimated resulting translation of a voxel on a 50 mm radius sphere. Following these operations, the magnitudes (absolute values) of all filtered MPs were summed to obtain low-pass-filtered framewise displacement (LPF-FD)[16,19]. For use as nuisance variables in the calculation of partial correlations, a set of motion parameters filtered by a 0.009 to 0.08 Hz second-order zero-phase Butterworth band-pass filter were also produced (fMPs).

Framewise BOLD signal fluctuation was calculated by first filtering all within-brain voxel time series using a 0.2 Hz zero-phase second order Butterworth filter. The temporal derivative (by backwards differences) was then calculated for each voxel, and the root-mean-square across all brain voxels of this derivative at each time point produced a single LPF-DV time series for each run[16,19-22].

Resting-State fMRI Volume Censoring

Motion denoising via volume censoring (scrubbing) was performed as described in prior work[16,19,22]. In summary, motion-contaminated volumes were identified using a fixed, study-wide LPF-FD threshold (Φ_F) maintained across all individuals, in tandem with run-specific LPF-DV thresholds (Φ_D) identified adaptively for each BOLD run (i.e., run-adaptive GEV-DV censoring)[22]. Additionally, any volume acquired while participants were recorded to have been closing their eyes was censored.

Within each run, the distribution of observed LPF-DV values were fit to a generalized extreme value (GEV) distribution[23] using maximum likelihood estimation, producing the run-specific shape parameter k_G. The empirical cumulative distribution function (ECDF) was then used to determine the run-specific LPF-DV threshold value Φ_D such that the ECDF at LPF-DV= Φ_D is equal to $1 - \frac{k_G+0.3}{d_G}$, where d_G is a study-wide free parameter[22].

Any volume with either an LPF-FD value exceeding the study-wide threshold (Φ_F), or an LPF-DV value exceeding the run-specific threshold (Φ_D), was excluded from analysis. Prior to band-pass filtering, censored time points were first replaced using linear interpolation. Subsequently, they were removed before calculation of partial correlations. Any run in which fewer than 150 volumes remained for the calculation of partial correlations due to excessive motion or eye closures was removed from analysis.

Study-wide parameters Φ_F and d_G were determined as described previously[22], using methods developed in the HCP 500 Subjects Release[5,6,24]. These were used to estimate optimal censoring thresholds in a multiband (high-TR) fMRI dataset, given a specified number of observations in each participant. The procedure was implemented in a publicly-available software release (https://www.mathworks.com/matlabcentral/fileexchange/73479-multiband_fmri_volume_censoring), after accounting for loss of runs and volumes due to eye closures. The study-wide number of runs was calculated as the harmonic mean of the number of runs acquired for each participant, after removing due to excessive eye closures any run in which fewer than 150 volumes remained for analysis (and after removing the first and last 30 volumes of each run). In each run k acquired during

session *j* for participant *i*, with 30 nuisance variables included in the partial correlation, this is equivalent to:

$$DoF_{i,j,k} = n_{V\,i,j,k} - 30 - 3 - n_{closures_{i,j,k}} \,,$$

where $n_{closures_{i,j,k}}$ is the number of volumes lost in run *k* of session *j* for participant *i* due to eye closure. The effective number of degrees of freedom across the entire study, DoF_{study}, is thus the harmonic mean of $DoF_{i,j,k}$ calculated iteratively across runs, sessions, and participants. For easy use with previously developed software, this can be converted to an effective number of volumes per participant:

$$n_{Vol_{Study}} = DoF_{study} + 30 + 3 \,.$$

This procedure produced an optimal Φ_F of 19.6085 mm and optimal d_G of 1.7781 (dimensionless), which were used as here as study-wide volume censoring parameters.

SUPPLEMENTARY RESULTS

Smoking Urges and Positive Cigarette Sensations

No effects with these variables were significant, though the active dTMS group reported marginally lower positive cigarette sensations and smoking urges than the sham group across Visits (Supplementary Table 1). In exploratory analyses, these overall group trends appeared more pronounced at Visit 17 than Visit 1. More specifically, participants in the active dTMS group reported fewer positive sensations of smoking (*b*=-1.36, *SE*=0.64, *p*=0.034) and marginally lower craving for cigarettes (*b*=-1.36, *SE*=0.72, *p*=0.060) than the sham group at Visit 17 (post-treatment), but not at Visit 1 (pre-treatment) (*p*s>0.39) (Supplementary Figure 1A-B). This pattern of effects requires additional exploration in future, though one possibility is that dTMS interrupted an

‘incubation of craving’ that naturally occurs when drug use is discontinued (or, in this case, reduced; see below)[25-27].

Daily Cigarettes Smoked

All participants, across both experimental conditions, reported smoking fewer cigarettes per day after the study than before it (Supplementary Figure 1C) (Supplementary Table 1), consistent with a quit attempt initiated during the study. This reduction amounted to 3.4 ± 0.9 fewer cigarettes per day, with a numerically (albeit not significantly) bigger reduction in the active dTMS group (-4.2 ± 1.4) compared with sham (-2.6 ± 1.0). Only one participant, in the sham group, reported full smoking cessation.

Negative and General Symptoms of Schizophrenia

We reported the results of positive symptoms in the main text, showing a stepwise decrease in symptoms over the course of the study in the active dTMS group. In contrast, such patterns were not observed for PANSS Negative or PANSS General. For negative symptoms, no effects or trends were detected (Supplementary Table 2). For general symptoms, Visit 0 was higher than the other four Visits (Supplementary Table 2). However, unlike for positive symptoms, the trajectory for general symptoms was quadratic (b=1.29, SE=0.31, p<0.001), such that symptoms dipped during the middle part of the study but began to re-emerge by its end. Given that this quadratic trajectory was observed for both the active dTMS (b=1.10, SE=0.38, p=0.003) and sham (b=1.48, SE=0.50, p=0.003) groups, and given that a plausible mechanism is elusive, we will not interpret these effects any further.

Left vs. Right Insula ASL

In the main text, we reported a main effect of ASL in the bilateral insula, such that blood flow during Visit 2 was lower than in Visit 1 ($b=-3.67$, $SE=1.72$, $p=0.033$). We further reported that Visit 2 differed from Visit 1 in the active dTMS group ($b=-4.11$, $SE=2.09$, $p=0.0495$) but not the sham group ($p=0.24$), further evidenced by a quadratic contrast which was significant in the active dTMS group ($b=1.86$, $SE=0.80$, $p=0.021$) but not the sham group ($p=0.51$). Here, we re-ran these same analyses, but this time separately for the left and right insula to inspect for any potential laterality effects of the dTMS (though none were predicted).

For the left insula, the main effect of insula blood flow (Visit 2 < Visit 1) was detected a trend level ($b=-4.01$, $SE=2.20$, $p=0.068$). This main effect was driven by the active dTMS group ($b=-5.18$, $SE=2.41$, $p=0.031$) rather than the sham dTMS group ($b=-2.84$, $SE=3.67$, $p=0.44$). Similarly, the quadratic contrast was significant in the active dTMS group ($b=2.30$, $SE=0.90$, $p=0.010$) but not the sham dTMS group ($b=0.68$, $SE=1.52$, $p=0.65$). This pattern of results is consistent with the bilateral ASL effects reported in the main text.

For the right insula, the main effect of insula blood flow (Time 2 < Time 1) was significant ($b=-3.35$, $SE=1.56$, $p=0.032$). Here, however, the active dTMS group and the sham dTMS group showed comparable reductions in insula ASL between Visits 2 and 1 (active dTMS: $b=-3.10$, $SE=2.18$, $p=0.16$; sham dTMS: $b=-3.60$, $SE=2.23$, $p=0.11$). The quadratic contrasts were also comparable in magnitude (active dTMS: $b=1.44$, $SE=1.08$, $p=0.18$; sham dTMS: $b=0.79$, $SE=0.93$, $p=0.39$). This pattern of results differs from the bilateral ASL effects.

Taken together, both the left and right insula showed the overall reduction in cerebral blood flow across the two treatment groups. Although there was some indication that the left insula showed a greater differentiation in the therapeutic effect, evidence for a laterality would need to be confirmed in future studies before firm conclusions can be drawn (particularly in the absence of a Time × Group interaction).

Supplementary References

1 Heatherton, T. F., Kozlowski, L. T., Frecker, R. C. & Fagerstrom, K. O. The Fagerstrom Test for Nicotine Dependence: a revision of the Fagerstrom Tolerance Questionnaire. *Br J Addict* **86**, 1119-1127 (1991).

2 Miller, W. R. & Del Boca, F. K. Measurement of drinking behavior using the Form 90 family of instruments. *J Stud Alcohol Suppl* **12**, 112-118 (1994).

3 Cox, L. S., Tiffany, S. T. & Christen, A. G. Evaluation of the brief questionnaire of smoking urges (QSU-brief) in laboratory and clinical settings. *Nicotine & tobacco research : official journal of the Society for Research on Nicotine and Tobacco* **3**, 7-16, doi:10.1080/14622200020032051 (2001).

4 Cappelleri, J. C. *et al.* Confirmatory factor analyses and reliability of the modified cigarette evaluation questionnaire. *Addict Behav* **32**, 912-923, doi:10.1016/j.addbeh.2006.06.028 (2007).

5 Glasser, M. F. *et al.* The Human Connectome Project's neuroimaging approach. *Nat Neurosci* **19**, 1175-1187, doi:10.1038/nn.4361 (2016).

6 Van Essen, D. C. *et al.* The WU-Minn Human Connectome Project: an overview. *Neuroimage* **80**, 62-79, doi:10.1016/j.neuroimage.2013.05.041 (2013).

7 Glasser, M. F. *et al.* The minimal preprocessing pipelines for the Human Connectome Project. *Neuroimage* **80**, 105-124, doi:10.1016/j.neuroimage.2013.04.127 (2013).

8 Robinson, E. C. *et al.* MSM: a new flexible framework for Multimodal Surface Matching. *Neuroimage* **100**, 414-426, doi:10.1016/j.neuroimage.2014.05.069 (2014).

9 Robinson, E. C. *et al.* Multimodal surface matching with higher-order smoothness constraints. *Neuroimage* **167**, 453-465, doi:10.1016/j.neuroimage.2017.10.037 (2018).

10 Smith, S. M. *et al.* Advances in functional and structural MR image analysis and implementation as FSL. *Neuroimage* **23 Suppl 1**, S208-219, doi:10.1016/j.neuroimage.2004.07.051 (2004).

11 Jenkinson, M., Beckmann, C. F., Behrens, T. E., Woolrich, M. W. & Smith, S. M. Fsl. *Neuroimage* **62**, 782-790, doi:10.1016/j.neuroimage.2011.09.015 (2012).

12 Fischl, B. FreeSurfer. *Neuroimage* **62**, 774-781, doi:10.1016/j.neuroimage.2012.01.021 (2012).

13 Grabner, G. *et al.* Symmetric atlasing and model based segmentation: an application to the hippocampus in older adults. *Med Image Comput Comput Assist Interv* **9**, 58-66, doi:10.1007/11866763_8 (2006).

14 Desikan, R. S. *et al.* An automated labeling system for subdividing the human cerebral cortex on MRI scans into gyral based regions of interest. *Neuroimage* **31**, 968-980, doi:10.1016/j.neuroimage.2006.01.021 (2006).

15 Fischl, B. *et al.* Automatically parcellating the human cerebral cortex. *Cereb Cortex* **14**, 11-22, doi:10.1093/cercor/bhg087 (2004).

16 Power, J. D. *et al.* Methods to detect, characterize, and remove motion artifact in resting state fMRI. *Neuroimage* **84**, 320-341, doi:10.1016/j.neuroimage.2013.08.048 (2014).

17 Jenkinson, M. & Smith, S. A global optimisation method for robust affine registration of brain images. *Med Image Anal* **5**, 143-156, doi:10.1016/s1361-8415(01)00036-6 (2001).

18 Jenkinson, M., Bannister, P., Brady, M. & Smith, S. Improved optimization for the robust and accurate linear registration and motion correction of brain images. *Neuroimage* **17**, 825-841, doi:10.1016/s1053-8119(02)91132-8 (2002).

19 Power, J. D., Barnes, K. A., Snyder, A. Z., Schlaggar, B. L. & Petersen, S. E. Spurious but systematic correlations in functional connectivity MRI networks arise from subject motion. *Neuroimage* **59**, 2142-2154, doi:10.1016/j.neuroimage.2011.10.018 (2012).

20 Smyser, C. D., Snyder, A. Z. & Neil, J. J. Functional connectivity MRI in infants: exploration of the functional organization of the developing brain. *Neuroimage* **56**, 1437-1452, doi:10.1016/j.neuroimage.2011.02.073 (2011).

21 Afyouni, S. & Nichols, T. E. Insight and inference for DVARS. *Neuroimage* **172**, 291-312, doi:10.1016/j.neuroimage.2017.12.098 (2018).

22 Williams, J. C. & Van Snellenberg, J. X. Motion denoising of multiband resting state functional connectivity MRI data: An improved volume censoring method. *bioRxiv*, 860635, doi:10.1101/860635 (2019). Preprint at https://www.biorxiv.org/content/10.1101/860635v6.external-links.html

23 Prescott, P. & Walden, A. T. Maximum likelihood estimation of the parameters of the generalized extreme-value distrubtion. *Biometrika* **67**, 723-724 (1980).

24 Smith, S. M. *et al.* Resting-state fMRI in the Human Connectome Project. *Neuroimage* **80**, 144-168, doi:10.1016/j.neuroimage.2013.05.039 (2013).

25 Parvaz, M. A., Moeller, S. J. & Goldstein, R. Z. Incubation of Cue-Induced Craving in Adults Addicted to Cocaine Measured by Electroencephalography. *JAMA Psychiatry* **73**, 1127-1134, doi:10.1001/jamapsychiatry.2016.2181 (2016).

26 Grimm, J. W., Hope, B. T., Wise, R. A. & Shaham, Y. Incubation of cocaine craving after withdrawal. *Nature* **412**, 141-142 (2001).

27 Abdolahi, A., Acosta, G., Breslin, F. J., Hemby, S. E. & Lynch, W. J. Incubation of nicotine seeking is associated with enhanced protein kinase A-regulated signaling of dopamine- and cAMP-regulated phosphoprotein of 32 kDa in the insular cortex. *Eur J Neurosci* **31**, 733-741, doi:10.1111/j.1460-9568.2010.07114.x (2010).

Supplementary Figure Caption

Supplementary Figure 1. Exploratory smoking variables of interest. (A) dTMS had an effect on cigarette craving, showing a trend difference between the groups after treatment but not before it. (B) dTMS had a similar effect on smoking pleasantness, showing a significant group difference after treatment but not before it. (C) All study participants significantly reduced their smoking over the course of the study, regardless of experimental condition, consistent with their attempt to quit during the study. We note, however, that the difference in smoking was numerically, though not significantly, higher in the active dTMS group.

Supplementary Table 1: dTMS effects on exploratory smoking variables: craving and smoking

Regressor	M_{diff} (*SE*)	*p* Value	Source of Effect or Trend
Cigarettes Smoked Per Day			
Visit (ref=Visit 1)			
Visit 17	-3.39 (0.85)	<0.001*	Visit 17 < Visit 1
Group (ref=Sham)			
Active dTMS	-0.11 (3.99)	0.979	NA
Visit × Group			
Visit 17 × Active dTMS	-1.64 (1.71)	0.337	NA
Craving for Cigarettes			
Visit (ref=Visit 1)			
Visit 17	-0.17 (0.48)	0.726	NA
Group (ref=Sham)			
Active dTMS	-0.83 (0.54)	0.122†[a]	Active dTMS numerically lower than Sham
Visit × Group			
Visit 17 × Active dTMS	-1.06 (0.96)	0.270	NA
Pleasant Cigarette Sensations			
Visit (ref=Visit 1)			
Visit 17	-0.08 (0.31)	0.795	NA
Group (ref=Sham)			
Active dTMS	-0.98 (0.60)	0.103†[a]	Active dTMS numerically lower than Sham
Visit × Group			
Visit 17 × Active dTMS	-0.76 (0.61)	0.214	NA

Note. *$p<0.05$, †$p<0.15$; [a]Further tested with posthoc comparisons on an exploratory basis, as explained in the main text.

Supplementary Table 2: Exploratory effects of dTMS effects on clinical symptoms of schizophrenia

Regressor	*b* (*SE*)	*p* Value	Source of Effect or Trend
PANSS Positive Symptoms			
Visit (ref=Visit 0)			
Visit 5	-1.85 (0.46)	<0.001*	Visit 5 < Visit 0
Visit 10	-1.25 (0.61)	0.042*	Visit 10 < Visit 0
Visit 16	-1.50 (0.49)	0.002*	Visit 16 < Visit 0
Group (ref=Sham)			
Active dTMS	-2.55 (2.18)	0.242	NA
Visit × Group[b]			
Visit 5 × Active dTMS	0.70 (0.93)	0.452	NA
Visit 10 × Active dTMS	-1.10 (1.23)	0.371	NA
Visit 16 × Active dTMS	-2.20 (0.99)	0.026*	Linear contrast (decrease over time) in Active dTMS but not Sham
PANSS Negative Symptoms			
Visit (ref=Visit 0)			
Visit 5	0.90 (1.01)	0.374	NA
Visit 10	-0.15 (1.35)	0.911	NA
Visit 16	1.55 (1.24)	0.213	NA
Group (ref=Sham)			
Active dTMS	0.10 (2.34)	0.966	NA
Visit × Group[c]			
Visit 5 × Active dTMS	-2.80 (2.02)	0.166	NA
Visit 10 × Active dTMS	-1.50 (2.69)	0.578	NA
Visit 16 × Active dTMS	-4.10 (2.49)	0.100‡	NA
PANSS General Psychopathology Symptoms			
Visit (ref=Visit 0)			
Visit 5	-4.90 (0.94)	<0.001*	Visit 5 < Visit 0
Visit 10	-3.85 (1.01)	<0.001*	Visit 10 < Visit 0
Visit 16	-3.60 (0.94)	<0.001*	Visit 16 < Visit 0
Group (ref=Sham)			
Active DTMS	-3.85 (2.32)	0.100†[a]	Active dTMS marginally lower than Sham
Visit × Group[d]			
Visit 5 × Active dTMS	1.20 (1.88)	0.523	NA
Visit 10 × Active dTMS	1.30 (2.03)	0.521	NA
Visit 16 × Active dTMS	1.00 (1.89)	0.596	NA

Note. *$p<0.05$, †$p<0.15$; ‡initially $p<0.15$, but not after accounting for multiple comparisons; [a]Further tested with posthoc comparisons on an exploratory basis, as explained in the main text. [b]Omnibus Visit × Group interaction for PANSS Positive Symptoms: $\chi^2(3)=5.77$, $p=0.124$; [c]Omnibus Visit × Group interaction for PANSS Negative Symptoms: $\chi^2(3)=3.71$, $p=0.294$; [d]Omnibus Visit × Group interaction for PANSS Positive Symptoms: $\chi^2(3)=0.56$, $p=0.907$. For descriptive purposes, the decrease across visits for positive symptoms was most notable for “conceptual disorganization” and “suspiciousness/persecution” items. PANSS=Positive and Negative Syndrome Scale.

Supplementary Figure 1

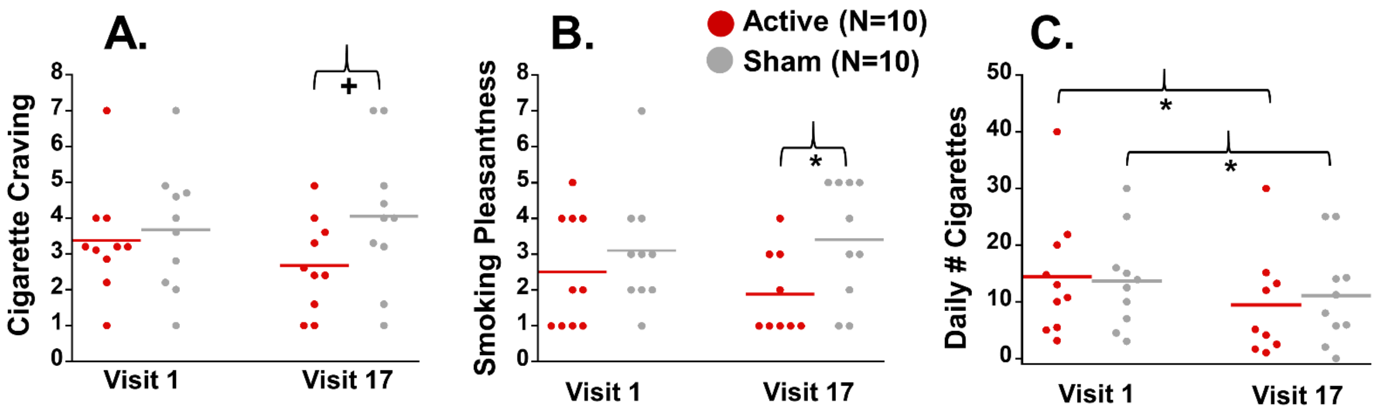

Supplement: Supplementary file 1 — Supplementary Material [file 41537_2022_224_MOESM1_ESM.pdf]
