# Supplementary material for: Deep rTMS of the insula and prefrontal cortex in smokers with schizophrenia: Proof-of-concept study
Source: Schizophrenia (Heidelb). 2022 Feb 25;8(1):6. doi: 10.1038/s41537-022-00224-0 (PMC8881463; doi:10.1038/s41537-022-00224-0)
Supplement: Supplementary file 2 — REPORTING SUMMARY [file 41537_2022_224_MOESM2_ESM.pdf]

nature portfolio

Corresponding author(s): Dr Scott Moeller

Last updated by author(s): 2021 12 01

Reporting Summary

Nature Portfolio wishes to improve the reproducibility of the work that we publish. This form provides structure for consistency and transparency in reporting. For further information on Nature Portfolio policies, see our Editorial Policies and the Editorial Policy Checklist.

Statistics

For all statistical analyses, confirm that the following items are present in the figure legend, table legend, main text, or Methods section.

n/a	Confirmed	
☐	☒	The exact sample size (*n*) for each experimental group/condition, given as a discrete number and unit of measurement
☐	☒	A statement on whether measurements were taken from distinct samples or whether the same sample was measured repeatedly
☐	☒	The statistical test(s) used AND whether they are one- or two-sided<br>*Only common tests should be described solely by name; describe more complex techniques in the Methods section.*
☐	☒	A description of all covariates tested
☐	☒	A description of any assumptions or corrections, such as tests of normality and adjustment for multiple comparisons
☐	☒	A full description of the statistical parameters including central tendency (e.g. means) or other basic estimates (e.g. regression coefficient) AND variation (e.g. standard deviation) or associated estimates of uncertainty (e.g. confidence intervals)
☐	☒	For null hypothesis testing, the test statistic (e.g. *F*, *t*, *r*) with confidence intervals, effect sizes, degrees of freedom and *P* value noted<br>*Give P values as exact values whenever suitable.*
☒	☐	For Bayesian analysis, information on the choice of priors and Markov chain Monte Carlo settings
☒	☐	For hierarchical and complex designs, identification of the appropriate level for tests and full reporting of outcomes
☐	☒	Estimates of effect sizes (e.g. Cohen's *d*, Pearson's *r*), indicating how they were calculated

Our web collection on statistics for biologists contains articles on many of the points above.

Software and code

Policy information about availability of computer code

Data collection	NA
Data analysis	Stata MP (v14.2)

For manuscripts utilizing custom algorithms or software that are central to the research but not yet described in published literature, software must be made available to editors and reviewers. We strongly encourage code deposition in a community repository (e.g. GitHub). See the Nature Portfolio guidelines for submitting code & software for further information.

Data

Policy information about availability of data

All manuscripts must include a data availability statement. This statement should provide the following information, where applicable:

- Accession codes, unique identifiers, or web links for publicly available datasets
- A description of any restrictions on data availability
- For clinical datasets or third party data, please ensure that the statement adheres to our policy

The data that support the findings of this study are available from the corresponding authors upon reasonable request.

Field-specific reporting

Please select the one below that is the best fit for your research. If you are not sure, read the appropriate sections before making your selection.

☒ Life sciences ☐ Behavioural & social sciences ☐ Ecological, evolutionary & environmental sciences

For a reference copy of the document with all sections, see nature.com/documents/nr-reporting-summary-flat.pdf

Life sciences study design

All studies must disclose on these points even when the disclosure is negative.

Sample size	This was a proof-of-concept study testing feasibility.
Data exclusions	There were no exclusions, except for brain imaging data due to poor quality.
Replication	There was no attempt at replication in this study.
Randomization	Participants were randomized to treatment conditions with stratification.
Blinding	The study was double-blind.

Reporting for specific materials, systems and methods

We require information from authors about some types of materials, experimental systems and methods used in many studies. Here, indicate whether each material, system or method listed is relevant to your study. If you are not sure if a list item applies to your research, read the appropriate section before selecting a response.

Materials & experimental systems

n/a	Involved in the study	
☒	☐	Antibodies
☒	☐	Eukaryotic cell lines
☒	☐	Palaeontology and archaeology
☒	☐	Animals and other organisms
☐	☒	Human research participants
☐	☒	Clinical data
☒	☐	Dual use research of concern

Methods

n/a	Involved in the study	
☒	☐	ChIP-seq
☒	☐	Flow cytometry
☐	☒	MRI-based neuroimaging

Human research participants

Policy information about studies involving human research participants

Population characteristics	Participants were smoking patients with schizophrenia, currently taking anti-psychotics. A full listing of the sample demographics is included in Figure 1B.
Recruitment	Participants were recruited from community clinics and hospitals in the greater New York area.
Ethics oversight	Stony Brook IRB.

Note that full information on the approval of the study protocol must also be provided in the manuscript.

Clinical data

Policy information about clinical studies

All manuscripts should comply with the ICMJE guidelines for publication of clinical research and a completed CONSORT checklist must be included with all submissions.

Clinical trial registration	As this study was not a full trial and instead was a feasibility study, we were not asked by the funding Institute to preregister.
Study protocol	As this study was not a full trial and instead was a feasibility study, we were not asked by the funding Institute to provide a study protocol.
Data collection	Participants were recruited in the greater New York Area. We screened 72 patients from June 2017 – January 2020, enrolling 32 of them.

Outcomes

All our outcome measures were pre-specified in the R21 mechanism that supported the study/

Magnetic resonance imaging

Experimental design

Design type

Resting-state; ASL

Design specifications

7.5 min, pre-treatment and post-treatment.

Behavioral performance measures

NA.

Acquisition

Imaging type(s)

Functional.

Field strength

3T

Sequence & imaging parameters

Four runs of multiband61 blood-oxygenation-level-dependent (BOLD) sensitive echo-planar imaging (EPI) T2*-weighted resting-state imaging were acquired per session. Each run comprised 563 volumes, approximately 7.5 min per run. During acquisition, participants fixated on a white cross. Phase encoding direction (i.e., anterior-posterior and posterior-anterior) was alternated for each run. The following acquisition parameters were used: multiband acceleration of 6 and no GRAPPA, 2mm isotropic, 204mm FOV, 66 slices, 60° FA, and TR/TE= 800/25ms (for complete information, see the Supplement);

3D-GRASE pseudo-continuous arterial spin labeling (pCASL) images were acquired with the following parameters: TR/TE/label time/post label delay (PLD)=4000/17.6/1600/1700ms, FA=120[illegible], matrix size=80[illegible]80[illegible]40, 3mm isotropic resolution, GRAPPA parallel imaging factor=2, phase-encoding direction segmentation factor=2, partition direction segmentation factor=3, and number of control-label averages=12. Two background suppression pulses were applied to suppress ~90% of static tissue signal. Equilibrium magnetization images were acquired with the same imaging parameters except TR=8000ms and no background suppression.

Area of acquisition

Whole-brain.

Diffusion MRI ☐ Used ☒ Not used

Preprocessing

Preprocessing software

Data were preprocessed using the following Human Connectome Project (HCP)62 Minimal Preprocessing Pipelines v4.2 (https://github.com/Washington-University/HCPpipelines) (for complete information, see the Supplement), smoothed with a 2mm FWHM Gaussian kernel;

ASL images were processed using SPM12 and in-house customized ASLtoolbox57. The processing pipeline consisted of: realignment for motion correction; segmentation of the T1w image into gray matter (GM), white matter (WM), and cerebrospinal fluid (CSF) probability maps; coregistration of the T1w image, GM, WM, and CSF probability maps to ASL images; smoothing with a 3-mm full-width at half maximum (FWHM) isotropic Gaussian kernel; CBF quantification using the single-compartment model58 with 3D partial volume correction59; and coregistration of the partial volume corrected CBF map to the T1w image for region-of-interest (ROI) analysis. ROIs were generated from the T1w images using FreeSurfer60.

Normalization

Extracted time series were mode 1000 normalized (multiplied by 1000 and divided of the modal value of all in-brain voxels), linearly detrended, and mean centered, followed by 0.009 to 0.08 Hz band-pass filtering using a second-order zero-phase Butterworth filter. After filtering, the first and last 30 volumes of each time series were discarded, due to contamination by edge artifacts produced by band-pass filtering.

Normalization template

Extracted time series were mode 1000 normalized (multiplied by 1000 and divided of the modal value of all in-brain voxels), linearly detrended, and mean centered, followed by 0.009 to 0.08 Hz band-pass filtering using a second-order zero-phase Butterworth filter. After filtering, the first and last 30 volumes of each time series were discarded, due to contamination by edge artifacts produced by band-pass filtering.

Noise and artifact removal

Extracted time series were mode 1000 normalized (multiplied by 1000 and divided of the modal value of all in-brain voxels), linearly detrended, and mean centered, followed by 0.009 to 0.08 Hz band-pass filtering using a second-order zero-phase Butterworth filter. After filtering, the first and last 30 volumes of each time series were discarded, due to contamination by edge artifacts produced by band-pass filtering.

Volume censoring

Motion denoising via volume censoring (scrubbing) was performed as described in prior work16,19,22. In summary, motion-contaminated volumes were identified using a fixed, study-wide LPF-FD threshold (ΦF) maintained across all individuals, in tandem with run-specific LPF-DV thresholds (ΦD)¬ identified adaptively for each BOLD run (i.e., run-adaptive GEV-DV censoring)22. Additionally, any volume acquired while participants were recorded to have been closing their eyes was censored. See supplement for more information.

Statistical modeling & inference

Model type and settings	ROI univariate. One analysis only per modality.
Effect(s) tested	Connectivity (time series correlation) between insula and default mode network; ASL-measured blood flow in bilateral insula.

Specify type of analysis: ☐ Whole brain ☒ ROI-based ☐ Both

Anatomical location(s)	Standard atlas.
Statistic type for inference (See Eklund et al. 2016)	NA. ROI only.
Correction	NA. ROI only.

Models & analysis

n/a	Involved in the study
☒	☐ Functional and/or effective connectivity
☒	☐ Graph analysis
☒	☐ Multivariate modeling or predictive analysis
